# Chemotactic responses of growing neurites to precisely controlled gradients of nerve growth factor

**DOI:** 10.1038/sdata.2018.183

**Published:** 2018-09-04

**Authors:** Brendan A. Bicknell, Zac Pujic, Julia Feldner, Irina Vetter, Geoffrey J. Goodhill

**Affiliations:** 1 Queensland Brain Institute, The University of Queensland, St Lucia, QLD 4072, Australia; 2 School of Mathematics and Physics, The University of Queensland, St Lucia, QLD 4072, Australia

**Keywords:** Axon and dendritic guidance, Developmental biology, Imaging

## Abstract

Chemotaxis plays a key role in many biological systems. In particular in the context of the developing nervous system, growing neurites can respond *in vitro* to shallow gradients of chemotropic molecules such as nerve growth factor (NGF). However, in such studies the gradient parameters are often not well controlled. Here we present a dataset of ~3500 images of early postnatal rat dorsal root ganglion (DRG) explants growing in 40 different precisely controlled combinations of absolute concentration and gradient steepness of NGF. Each image has been segmented into neurite and explant-body regions. We provide computer code for exploration and quantification of the data, including a Fourier analysis of the outer contour of neurite growth, which allows quantities such as outgrowth and guidance as a function of concentration and gradient steepness to be easily extracted. This is the most comprehensive quantitative dataset of chemotactic responses yet available for any biological system, which we hope will be useful for exploring the biological mechanisms governing chemotaxis.

## Background & Summary

Brain function relies on precise wiring between neurons. During neural development, growing neurites use a variety of molecular and mechanical cues to find their appropriate targets^1,2^. Much interest has focused on neurite chemotaxis, i.e. growing up or down molecular concentration gradients. While it is still believed that this is a key strategy for guiding axons *in vivo*, this idea has also recently begun to generate controversy^3–5^. However, it is certainly true that neurites *in vitro* show chemotactic responses to many of the molecules present in the developing nervous system^6–8^.

A key assay for studying chemotactic responses of neurites *in vitro* is to grow tissue explants in a collagen gel in the presence of gradients and look for asymmetric outgrowth of neurites from the explant. This provides an environment closer to that existing *in vivo* than assays examining chemotactic responses of dissociated neurons (e.g. ref. 9). In the collagen gel ‘co-culture’ assay the source of the chemotropic factor is an explant of the target tissue, or a block of cells expressing the factor, which set up a gradient by diffusion. However, the parameters of this gradient are difficult to precisely determine^10^.

**Figure 1:**
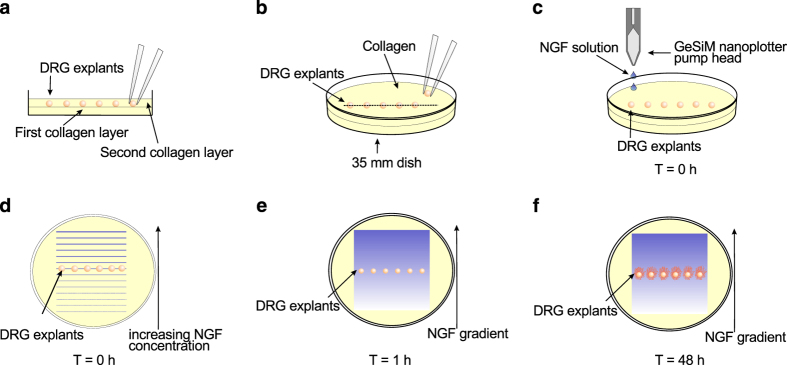
Summary schematic of the collagen gel assay for assessing neurite growth and guidance in shallow chemical gradients. (**a**,**b**) A single row of DRG explants was positioned in collagen gel in the centre of a 35 mm dish. (**c**,**d**) After the collagen had set, stock solutions with increasing NGF concentration were printed in parallel lines onto the surface with a GeSiM nanoplotter. (**e**) The printed lines of NGF rapidly diffused into the gel, forming a smooth exponential gradient that remained stable for many hours at the location of the explants. (**f**) The dish was incubated at 37° C for 40–48 h before fixation and measurement of neurite outgrowth. Reprinted from ref. 16 with permission from Journal of Neurotrauma, published by Mary Ann Liebert, Inc., New Rochelle, NY.

A technology that overcomes this limitation involves printing gradients of chemotropic factors onto the surface of a collagen gel, from where they diffuse into the body of the gel to produce a smooth gradient that can remain relatively stable for many hours^11,12^ (Fig. 1). Using this assay it was demonstrated that guidance of neurites by NGF gradients from DRG explants is only effective over a relatively narrow range of concentrations^13^, and that biased outgrowth in gradients is a form of chemotactic response rather than being a purely trophic effect^14^. These experiments involved accumulating a dataset of ~3500 images of fluorescently labelled DRG explants growing under 40 different precisely controlled NGF gradients of a range of concentrations and steepnesses. This is the most comprehensive dataset of chemotactic responses as a function of steepness and concentration yet performed for any biological system.

**Figure 2:**
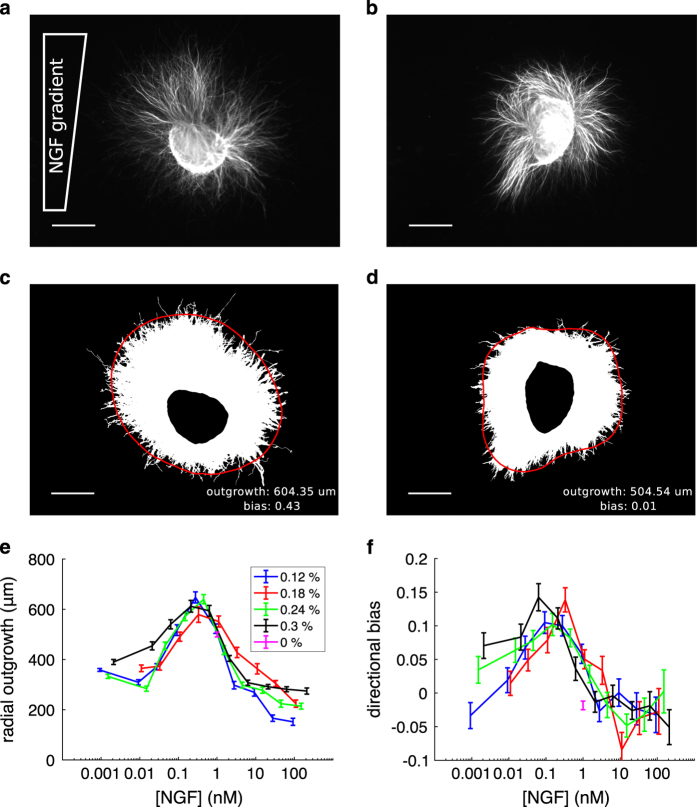
Example images and analysis. (**a**,**b**) Raw images of DRG explants (filenames ‘27_0806d_0p2_003p000.tif’ and ‘24_2506d_0p3_003p000.tif’ for **a** and **b**, respectively). The NGF gradient increases towards the top of the image. Scale bars 500 um. (**c**,**d**) Binary masks for the neurite regions of the images directly above. Superimposed is the Fourier approximation of radial outgrowth using five coefficients (red curve), and the outgrowth and bias measurements (text; lower right). Scale bars 500 um. (**e**) Quantification of average radial outgrowth by Fourier coefficient *a*_0_ as a function of background concentration (x-axis) and gradient steepness (colours; see legend). (**f**) Quantification of outgrowth bias up the gradient by normalised coefficient *b*_1_/*a*_0_ Error bars are SEM.

Here we present this dataset of raw and segmented images, along with a method of analysis for extracting quantitative information from them (see Fig. 2 for examples). Associated with each raw image file are a neurite mask file, identifying the region of neurite growth, and an explant mask file, giving the region comprising the cell bodies. A data structure and accompanying code is provided that contains the coordinates of the boundaries of the two regions, quantification of growth and guidance in terms of both area and length measurements, and higher-order shape properties via a Fourier decomposition. Moreover, the raw images also permit many other forms of analysis of chemotactic responses to be performed, with the potential to give fresh insight into the behaviour of both growing neurites and biological chemotaxis more generally.

## Methods

### Explant preparation

The following is a summary of methods for explant preparation detailed in previous work^11,13^. DRGs were removed from the thoracic and lumbar regions of postnatal day P0-P3 rat pups and trimmed of excess tissue. Explants were stored in Hibernate E (phenol red; Brainbits) at 4° C overnight. On the next day, the connective tissue sheath was loosened by incubation for 12 min in 0.25% trypsin/10 ug/mL DNase1/Ca^2+^ and Mg^2+^ free Hank’s balanced salt solution. The explants were centrifuged and resuspended in Leibovitz’s L-15 medium containing L-glutamine and 0.45% D(+)-glucose 3 times.

A 0.2% collagen gel solution was prepared on ice with the following concentrations: rat tail type I collagen stock solution (BD Biosciences) diluted with water to contain 0.2 mg/mL collagen, 27 µL of a 7.5% sodium bicarbonate solution per mL of original collagen stock, 1×OptiMEM (Gibco), 100 µg/mL penicillin, 100 µg/mL streptomycin, and 250 µg/mL amphotericin (Gibco). 750 µL of collagen was spread uniformly over the bottom of a 35 mm tissue culture dish and allowed to set. A second 750 µL layer of collagen was added, and 6 explants arranged in a line in the centre of the dish before the collagen set. The dishes were incubated for 15 min to allow setting before gradient printing (Fig. 1a and b).

### Shallow gradient assay

Gradients of NGF (GroPep) were created by using a Nano-Plotter 2.0 (Gesim). Twelve stock solutions with exponentially increasing NGF concentration were ‘printed’ onto the surface of the collagen gels in the form of 12 parallel lines 20 mm long and 1 mm apart, each line containing the same volume of stock. Line 4 coincided with the position of the row of explants. The amount of NGF required in each line to produce the desired final concentration in the gel was calculated as described in refs 11, 12. However, in ref. 13 we also provided correction factors for both the concentration and gradient steepness, to take into account the average gradient conditions existing over the complete time course of the experiment. Four additional ‘pregradient’ lines of only vehicle (0.1% BSA/PBS) were applied adjacent to the low-concentration side of the gradient (line 1) to avoid a possibly confounding gradient of collagen density near to the explants. After printing, dishes were returned to a 37° C incubator with 5% CO_2_ for a total explant incubation time of 40–48 h. Our standard control was to print a ‘plateau’ by using the same methods, except with no change in NGF concentration between the different stocks (Fig. 1c-f).

### Immunostaining and microscopy

Explants embedded in collagen were fixed with 10% formaldehyde/0.1% Triton-X 100 in PBS overnight. Plates were washed five times with PBS with 1 hour between washes, and then incubated overnight at 4° C with *β*-III-tubulin antibody TuJ1 (1:500; R&D Systems). After five washes with PBS of 1 hour each, plates were incubated overnight at 4° C with secondary antibody Alexa Flour 488-conjugated goat anti-mouse IgG (1:1000, Molecular Probes). Plates were washed five times in PBS for 1 hour each before acquisition of images with an AxioCam HRm (Zeiss) camera on a Zeiss Z1 fluorescence microscope.

### Quantification of neurite outgrowth

Images were manually segmented (using Adobe Photoshop or ImageJ) to separate the cell-body and neurite outgrowth regions, and thresholded by pixel intensity to form binary masks. The values of threshold pixel intensity, which differed among images depending on background intensity, were selected conservatively to avoid spurious contributions from image noise. In the neurite outgrowth mask, the region of connected non-zero pixels of the thresholded image that was contiguous with the explant body region was retained, and the remaining disconnected components were removed. The resulting binarised images provide reduced representations of the explants from which key area and length-based measurements can be easily extracted by automated analysis. For further analyses that also incorporate features such as neurite density, the provided image masks can be used to select the relevant regions of interest in the corresponding raw images.

Area-based measurements of neurite outgrowth and guidance, as used by refs 11,^13^,^14^, were calculated by counting the number of non-zero pixels in the binary masks. Outgrowth (OG) was quantified by the number of non-zero pixels in the neurite mask, divided by the number in the explant-body mask. Guidance was quantified by the ‘guidance ratio’ $\mathrm{GR}=\frac{H-L}{H+L}$, where H and L are the number of non-zero pixels in the high (H) and low (L) concentration sides of the neurite mask, with respect to the midpoint defined by the centroid of the cell-body mask.

To characterise the growth response in greater detail, a Fourier analysis of radial outgrowth was introduced in ref. 15. Boundary curves were fitted to the cell-body mask and the outer boundary of the outgrowth mask using the MATLAB function ‘bwboundaries’. The curves were smoothed with a moving average filter of width 150 pixels, and then parameterised by polar coordinates with *N*=360 discrete angles $\theta_{n}=\frac{2\pi n}{N}$ about an origin defined as the centroid of the cell-body mask. In the event that a ray from the origin intersected a boundary at multiple points, the closest point to the origin was selected. The radial outgrowth function, *R*(*θ*_*n*_), was defined as the distance between the cell-body and neurite region boundaries at each *θ*_*n*_. We extended this to a continuous representation by performing a discrete Fourier transform to give *R*(*θ*_*n*_) in terms of frequency components, $R(\theta_{n})=\sum_{k=0}^{N-1}{\hat{R}}_{k}\cdot e^{2\pi ikn/N}$, and then folding about the Nyquist frequency to determine equivalent Fourier coefficients as a0=Re(Rˆ0), ak=2Re(Rˆk) and bk=−2Im(Rˆk), 1≤k≤180.

We found that the first five coefficients were sufficient to reconstruct the major features of explant outgrowth via

(1)R(θ)≈a0+a1cos(θ)+b1sin(θ)+a2cos(2θ)+b2sin(2θ),

as demonstrated in the examples in Fig. 2c and d. The coefficient *a*_0_ determines the average radial outgrowth, *a*_1_ and *b*_1_ determine the bias in outgrowth in orthogonal image axes, and *a*_2_ and *b*_2_ capture the polarised growth exhibited by some explants. In Fig. 2e we use the coefficient *a*_0_ to quantify average outgrowth (in units of *um*), and in Fig. 2f we use the normalised coefficient *b*_1_/*a*_0_ as a dimensionless measure of outgrowth bias up the gradient.

### Code availability

MATLAB code for displaying images and their segmentation, as well as the data analysis and figures presented here is provided with the data. Code for fitting the explant boundaries and computing outgrowth and guidance measurements is available at https://github.com/babicknell/NeuriteGrowth. Code was written using MATLAB version 2017b, and is compatible with earlier versions (version 2015b tested).

## Data Records

The provided directory (Data Citation 1) contains a data set of segmented images of dorsal root ganglia explants grown in NGF gradients, and MATLAB files for quantification and analysis.

**images** This subdirectory contains the raw images. Each raw image is a tif file of size ~1MB. The first two digits of a filename specify the experiment number (28 in total), the second two digits specify the plate number within that experiment, and the third two digits specify the explant number within that plate. The following letter ‘u’ (up) or ‘d’ (down) records the orientation of the plate at which the image was captured. All ‘d’ type images have been rotated by 180 degrees so that for all images in the data set the NGF gradient, when present, is increasing towards the top of the image. The remaining digits specify the gradient steepness (% concentration change per 10 um) and background concentration (nM). For example, ‘0p4’ means ‘zero point four’. As a complete example, ‘03_0704d_0p3_000p100.tif’ denotes experiment number 3, plate 7, explant 4, with gradient steepness 0.3% change per 10 um, and a 0.1 nM background concentration. The scale factor for converting image pixels to physical length is 2.58 um per pixel.

**neurite_masks** This subdirectory contains the manually segmented binary masks demarcating the region of neurite outgrowth. Filenames are as above, with the additional suffix ‘n’.

**explant_masks** This subdirectory contains manually segmented binary masks demarcating the central cell-body region of the explant. Filenames as above, with the additional suffix ‘x’.

## MATLAB files

To access the MATLAB content, the parent directory should be opened within MATLAB, and ‘S.mat’ loaded into the workspace. Functions can be called from the command line as follows.

Growth and guidance plots: plot_growth_guidance(S)

Display eg. image 2269: display_image(S,2269)

Plot eg. Fourier coefficient *a*_1_: plot_coeff(S,’a1’)

**S.mat** This MATLAB data structure contains the coordinates of the boundaries of the relevant regions in the binary mask images, and quantification of explant outgrowth. The structure contains the following fields.

‘name’: filename of image.

‘gradient’: gradient steepness (% change per 10 um).

‘concentration’: background concentration (nM).

‘outgrowthBoundary’: *N*×2 matrix containing *N* cartesian coordinates of the boundary of neurite outgrowth.

‘somataBoundary’: *M*×2 matrix containing *M* cartesian coordinates of the boundary of the central cell-body region.

‘centroid’: centroid of the cell-body region in the image. Used for plotting boundary curves over the original image (see display_image.m).

‘ougrowthCoeffs’: 181×2 matrix of Fourier series coefficients for radial outgrowth function (distance between outgrowth and cell-body region boundaries, parameterised in polar coordinates). The first column contains the cosine coefficients *a*_*n*_, the second column contains the sine coefficients *b*_*n*_.

‘somataCoeffs’: 181×2 matrix of Fourier coefficients for cell-body region only.

‘averageOutgrowth’: average radial outgrowth of the explant, quantified by outgrowth coefficient *a*_0_.

‘directionalBias’: bias in outgrowth up the gradient, quantified by normalised outgrowth coefficient *b*_1_/*a*_0_.

‘OG’: (number of non-zero neurite mask pixels)/(number of non-zero explant mask pixels).

‘GR’: Guidance Ratio (number of up-gradient neurite pixels minus number of down-gradient neurite pixels)/(total number of neurite pixels).

**IND.mat**: This MATLAB data structure is a 5×10 cell array of indices of data structure ‘S’ corresponding to each experimental condition. For gradients steepnesses *s*=[0, 0.1, 0.2, 0.3, 0.4] and concentrations *c*=[0.001, 0.01, 0.03, 0.1, 0.03, 0.1, 0.3, 1, 3, 10, 30, 100], the vector stored in IND{*i,j*} gives the indices for condition *s*(*i*),*c*(*j*). Eg. IND{1,6} is a 1×455 vector of indices *k* such that *S*(*k*) is a file from the 0% gradient, 1 nM concentration control condition.

**display_image.m** This MATLAB function displays a given image with the boundary curves and Fourier approximation (default 5 components) superimposed. ‘S.mat’ must first be loaded into the workspace, and then the function called with the syntax ‘display_image(S,file)’, where ‘file’ can either be an index of S, or a string corresponding to a given file (eg. ‘03_0704d_0p3_000p100’).

**plot_growth_guidance.m** This MATLAB function produces plots of the concentration and gradient dependence of outgrowth, using both the Fourier and pixel count methods. Load ‘S.mat’, and call with ‘plot_growth_guidance(S)’. The correction factors detailed in ref. 13 (variable ‘cf’ within the function) are applied to adjust the average concentration for each gradient condition before plotting.

**plot_coeff.m** This MATLAB function plots the concentration and gradient dependence of a given Fourier coefficient, normalised by average outgrowth *a*_0_. Load ‘S.mat’, and then call with ‘plot_coeff(S,coeff)’, where ‘coeff’ is a string of the form ‘an’ or ‘bn’. Eg. calling with coeff=‘a1’ plots the normalised cosine coefficient representing outgrowth bias orthogonal to the gradient direction.

## Technical Validation

The accuracy and stability of the gradient produced by the printing assay was validated by quantitative imaging of a fluorescently labelled marker, and finite element simulations, as detailed in ref. 11. Accurate segmentation of the images was confirmed by eye, and can also be directly validated by the user with the code provided (‘display_image.m’). Total outgrowth and bias in outgrowth as a function of NGF concentration and gradient steepness were previously presented based on counting pixels representing neurites^13^. The provided code ‘plot_growth_guidance.m’ shows almost identical curves generated from both the pixel counting and boundary contour methods.

## Additional information

**How to cite this article**: Bicknell, B. A. *et al*. Chemotactic responses of growing neurites to precisely controlled gradients of nerve growth factor. *Sci. Data* 5:180183 doi: 10.1038/sdata.2018.183 (2018).

**Publisher’s note**: Springer Nature remains neutral with regard to jurisdictional claims in published maps and institutional affiliations.

## Supplementary Material
